# Application of Sol–Gels for Treatment of Gynaecological Conditions—Physiological Perspectives and Emerging Concepts in Intravaginal Drug Delivery

**DOI:** 10.3390/gels8020099

**Published:** 2022-02-08

**Authors:** Ritu Thapa, Shila Gurung, Marie-Odile Parat, Harendra S. Parekh, Preeti Pandey

**Affiliations:** 1 School of Pharmacy, The University of Queensland, 20 Cornwall St, Woolloongabba, QLD 4102, Australia; r.thapa@uqconnect.edu.au (R.T.); m.parat@pharmacy.uq.edu.au (M.-O.P.); 2 School of Health and Allied Sciences, Pokhara University, Pokhara-30, Kaski 33700, Nepal; gshila@gmail.com

**Keywords:** vaginal drug delivery, sol–gel formulations, stimuli-responsive polymers, mucoadhesion, vaginal applicators/devices

## Abstract

Approaches for effective and sustained drug delivery to the female reproductive tract (FRT) for treating a range of gynaecological conditions remain limited. The development of versatile delivery platforms, such as soluble gels (sol–gels) coupled with applicators/devices, holds considerable therapeutic potential for gynaecological conditions. Sol–gel systems, which undergo solution-to-gel transition, triggered by physiological conditions such as changes in temperature, pH, or ion composition, offer advantages of both solution- and gel-based drug formulations. Furthermore, they have potential to be used as a suitable drug delivery vehicle for other novel drug formulations, including micro- and nano-particulate systems, enabling the delivery of drug molecules of diverse physicochemical character. We provide an anatomical and physiological perspective of the significant challenges and opportunities in attaining optimal drug delivery to the upper and lower FRT. Discussion then focuses on attributes of sol–gels that can vastly improve the treatment of gynaecological conditions. The review concludes by showcasing recent advances in vaginal formulation design, and proposes novel formulation strategies enabling the infusion of a wide range of therapeutics into sol–gels, paving the way for patient-friendly treatment regimens for acute and chronic FRT-related conditions such as bacterial/viral infection control (e.g., STDs), contraception, hormone replacement therapy (HRT), infertility, and cancer.

## 1. Introduction

Recent advances in pharmaceutical research and development have drawn considerable attention towards developing more effective, patient-friendly, and clinician-endorsed treatment options for conditions of the FRT. Compared to the oral/parenteral routes, local and direct vaginal drug delivery (VDD) is preferred for both small and large molecules, owing to the mucosal surface area of the upper and lower FRT, with its rich blood supply, and avoidance of the gastrointestinal tract and hepatic first-pass metabolism. From the patients’ perspective, VDD minimises off-target effects and enables self-administration in the case of lower FRT conditions, obviating the need for specially trained medical personnel [1,2,3].

The vagina offers a direct channel for delivering therapeutic agents for the treatment of various gynaecological conditions that have, to date, been exclusively delivered via the oral/systemic route. However, effective VDD is challenged by the highly variable anatomical, physiological, and microbiological features of the FRT, which transition over a patient’s lifetime, consequently posing a great challenge to formulation scientists where catering to the varied needs of patients can be effectively met.

In this regard, various conventional formulations (e.g., creams and pessaries), although administered vaginally, result in limited delivery success, due to the physiological clearing mechanism of the vagina, which results in premature leakage and sub-therapeutic outcomes. These shortfalls stem from a short drug residence time on mucosa, with lowered drug absorption and unfavourable clinical outcomes [1,4,5,6]. Thus, an alternative vehicle that can tolerate a changing microenvironment and has good mucoadhesive/retention properties, while having appropriate flow properties to adequately disperse, reach, and coat target mucosa/structures in the FRT, is sought.

In this context, phase-transforming sol–gels offer a safe, practical, economical, and effective solution to the long-standing issues hampering drug delivery to the upper and lower FRT [1]. Sol–gels are initially in solution form, enabling ease of dispersion onto mucosa with an appropriate device, undergoing rapid gelation in response to physiological stimuli such as body temperature, changes in pH, and/or the presence of counter ions. Once gelled, the mucoadhesive properties of an appropriately engineered sol–gel pave the way for the sustained delivery of infused therapeutics (e.g., small molecules, peptides, proteins, genes) to underlying mucosal tissue [7,8]. Additionally, the hybridisation of sol–gels with drug encapsulation strategies such as nanoparticles, microspheres, liposomes, and PEGylation can be used to further enhance/fine-tune the drug release characteristics [9]. Several studies confirm the successful delivery of drugs using sol–gel formulations in a number of FRT-related conditions, including infection treatment, the delivery of contraceptives, labour induction, supplementation of the microbiome, HRT, and prophylaxis of sexually transmitted diseases [10,11,12,13,14,15,16]. Given its immense potential, the highly versatile sol–gel platform technology has drawn great attention and interest from the scientific community in reimagining drug delivery to the FRT.

To this end, this review provides a comprehensive analysis of sol–gel systems to achieve the desired therapeutic outcomes for a range of gynaecological conditions that have not yet been effectively addressed using conventional dosage forms. It also highlights the various stimuli-responsive sol–gel systems and discusses the emerging advanced drug encapsulation technologies that can be incorporated into sol–gels to develop an “ideal” vaginal drug delivery system (VDDS). Furthermore, it discusses the use of “smart polymers” to create site-specific, FRT-responsive sol–gel systems factoring in physiological and pharmaceutical considerations. The review preferentially highlights published works relating to VDD since 2010, while a selected few seminal works pre-dating this timeframe were also included. The key databases used to gather material for this review were Science Direct, PubMed, and Web of Science, with only peer-reviewed articles published exclusively in English included.

## 2. Anatomical and Physiological Features of the Female Reproductive Tract

**Figure 1:**
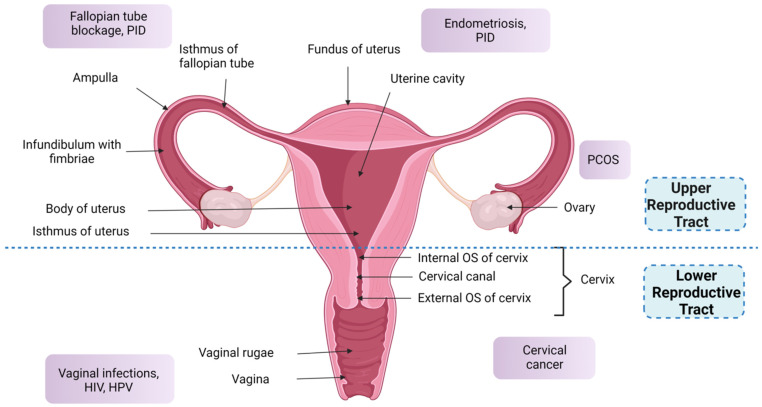
Schematic representation of key anatomical features of the upper and lower FRT, with the associated gynaecological conditions shown in purple boxes. PID—pelvic inflammatory disease, PCOS—polycystic ovarian syndrome, HIV—human immune deficiency virus, HPV—human papilloma virus.

The organs of the FRT can be classified as residing in the upper reproductive tract, which includes the ovaries, uterus, and fallopian tubes, and the lower reproductive tract, which includes the cervix and vagina, as depicted in Figure 1 [17].

The dimensions, key features, and conditions of the FRT are summarised in Table 1. As is evident in Table 2, the pH shift from acidic in the vagina to alkaline in the upper parts of the FRT, variation in the volume and consistency of cervicovaginal mucus, and the microbial content make the physiological features of FRT highly variable. These changing dynamics of the FRT remain a major challenge in effective drug delivery but adequately engineered drugs can successfully overcome this barrier [18]. Thus, understanding the key anatomical and physiological features of the FRT is essential in order to design DDS through the highly challenging FRT [19,20].

**Table 1:** Anatomical features, functions, and associated conditions of the female reproductive tract.

	Site	Dimensions and Features	Functions	Associated Diseases	References
Upper reproductive tract	Uterus	7.5 cm length; 5 cm width; comprises fundus, body, and isthmus; uterine wall comprises endometrium (epithelial cells), myometrium (smooth muscle cells), and perimetrium (connective tissue)	Implants, nourishes, and protects the embryo; receptors for sex steroids present in endometrium	Adenomyosis; Endometriosis; Luteal phase defect; Uterus fibroids	[21,22,23,24]
	Fallopian tubes	7–14 cm length; lumen is 0.1–1 mm in uterus–isthmus junction, 1–2 mm in isthmus–ampullary junction, and 1 cm in ampulla–infundibulum junction; comprises isthmus, ampulla, and infundibulum; tube wall composed of endosapinx (innermost mucosal layer), myosalpinx (middle muscular layer), and external serosa	Ciliated cells mobilise gametes and embryo; secretory cells nourish oocyte and embryo	Tubular blockage	[25,26]
	Ovaries	2.5–3.5 cm length; 2 cm width; comprises internal medulla and external cortex consisting of follicles and stroma	Release oocytes, estrogen, and progesterone	Polycystic ovarian syndrome	[26,27]
Lower reproductive tract	Cervix	3–4 cm length; 2.5 cm width; comprises endocervix (columnar cells) and ectocervix (squamous epithelium cells); 20–60 mL/day of cervical mucus secretion pH 7.0; Mucus composition: 95–99% water, 1–4% enzymes/ proteins/ mucin	Acts as passage for sperm; supports foetus till birth faciliates childbirth; defensive roles against pathogens due to mucin and immunoglobulins	HPV infection; cervical cancer	[28,29,30]
	Vagina	7–15 cm length; 2.1–4.5 cm width; daily vaginal secretions ≈6 mL and 0.5–0.75 mL present at any given time;wider near the cervix; variable surface area of 50–600 cm^2^; transverse folds (rugae) present in vaginal wall; pH 3.8–4.2; vaginal wall comprises 30–100 µm thick mucus layer, layer of epithelium cells, lamina propria, muscular layer, and tunica adventitia	Rugae increases vaginal surface area and causes vaginal extension during coitus and childbirth; passage for menstrual flow and childbirth; acidic pH has defensive role against pathogens	Vaginal infection, atrophy, and lesions	[3,31]

**Table 2:** Microenvironmental conditions of the uterus, cervix, and vagina in healthy, non-pregnant individuals.

Site	Physiological Condition	pH	Wall Thickness (mm)	Mucus		Microbial Content	Microbial Diversity	References
				Volume or Mass (Daily)	Viscosity			
Uterus (Endometrium)	Follicular (proliferative)	7.22	4.0–10.0	-	-	-	-	[26,32]
	Ovulatory	7.35	10.0–11.0	1.5 mL	-	-	-	[26,32]
	Luteal (secretory)	7.0–7.8	4.0–6.0	-	-	-	-	[23,33,34]
Cervix	Follicular	<7.0	-	20–60 mg	High	-	-	[32,35,36]
	Ovulatory	7.0	-	700 mg	Low	-	-	[35,36,37]
	Luteal	<7.0	-	20–60 mg	High	-	-	[32,35,38]
Vagina	Pre-puberty	7.0	˂0.15	-	-	Low	High	[39,40]
	Follicular	4.0–6.0	0.075–1.0	4.14 g	High	High	Low	[6,26,31,41]
	Ovulatory	3.8–4.2	0.15–2.0	5.88 g	Low	High	Low	[6,31,37,39,40]
	Luteal	3.8–4.2	˂0.15	4.11 g	High	High	Low	[6,26,31,37]
	Menopause	6.0–7.5	0.11–0.15	<2.94 g	-	Low	High	[31,39,40,42,43]

Recently, a comprehensive review was published that focused on the scope of various female specific drug delivery routes including drug delivery to entire parts of the FRT transvaginally or by other means such as laparoscopy, abdominal puncturing, and ultrasound imaging [19]. While drug delivery to the FRT is discussed in our review, the focus herein is on the use of the emerging sol-gel technology, while highlighting the shortfalls of commercially available conventional vaginal dosage forms in the treatment of gynaecological conditions associated with distinct sites/tissues of the FRT. Furthermore, this review discusses the scope of this novel formulation technology in developing more clinically effective, economical, and patient-friendly drug delivery approaches for the FRT; this we propose can be achieved by exploiting the advantages of its challenging physiology alongside complementary formulation strategies, such as merging nanotechnological approaches and mucoadhesive drug delivery systems.

### 2.1. Upper Reproductive Tract

- Uterus

The uterus (Figure 1), a pear-shaped hollow organ, is affixed in the pelvic region and connects to two fallopian tubes at lateral ends. The tubes enter either side of the fundus, and the uterine body narrows downwards to the isthmus and finally leads to the cervix. It is nourished by the uterine artery (UA), which also supplies blood to the upper vagina. The UA arises from the internal iliac artery, which descends to the isthmus and anastomoses, which encompass the ovarian artery. The uterine vein drains into internal iliac veins and also connects to the ovaries, vagina, and bladder through a plexus of veins [22]. The uterine wall thickness changes throughout the monthly cycle and with age, which is highlighted in Table 2.

- Fallopian tubes

A pair of fallopian tubes (Figure 1) run medially from the uterus to the lateral side of the ovaries and end at the fimbriae, whose luminal diameter varies along the tube length, as mentioned in Table 1. These tubes are enriched with primary folds and contain ciliated, secretory cells and peg cells in the endosalpinx region, with a higher abundance of folds towards the distal portion and the presence of secondary folds in the ampulla and infundibulum regions [25].

- Ovaries

They are a pair of oval-shaped organs (Figure 1) attached to the pelvis by suspensory ligaments, and to the uterus by utero-ovarian ligaments. The cortex region of each ovary comprises follicles containing oocytes (eggs), which are released monthly upon maturation during ovulation, alongside follicular cells containing oestrogen. After ovulation, when the egg is released, the remaining follicle, known as the corpus luteum, secretes progesterone until a new pregnancy is established. However, if the pregnancy is not established, it regresses in size and becomes dysfunctional, resulting in diminished production of oestrogen and progesterone hormones and secretion of gonadotropin-releasing hormone (GnRH), follicle-stimulating hormone (FSH), and luteinising hormone (LH) to begin the development of new follicles for a new cycle. The medullary region is supplied with blood vessels, nerves, and fibrous tissues. Similarly, deeper parts of the ovaries contain Leydig cells, which secrete testosterone, dehydroepiandrosterone, and androstenedione [26,27].

### 2.2. Lower Reproductive Tract

- Cervix

The cervix (Figure 1) is a continuation of the uterus, connecting the uterine cavity with the vagina, which leads to the uterus by an internal orifice (internal OS), and to the vagina by an external orifice (external OS) through the ectocervix. The endocervical canal forms a passage between the internal and external OS and contains mucus of varying consistency, volume, and pH (detailed in Table 2), which originates both in the uterus and cervix and flows down to the vagina [35]. The acid–base transport mechanism present in uterus and cervix is responsible for the alkaline pH of the cervical fluid given that the abundance of lactobacilli is minimal in the cervical canal [34]. A squamocolumnar junction is present between the columnar cells of the endocervix and squamous cells of the ectocervix, which shifts proximally with the conversion of columnar cells into squamous cells. The junction is a major site of development of neoplasia in response to increased oestrogen levels and infections by human papilloma virus (HPV). It is nourished by the UA arising from the internal iliac artery [29,30].

- Vagina

It is an S-shaped organ descending from the cervix to the vestibule of the vagina (Figure 1). Although the vagina is devoid of secretory glands, the vaginal wall contains a thin mucus layer commonly known as vaginal fluid, which is composed mainly of water and mucin. This fluid is a mixture of secretions from the Bartholin gland and vestibular glands, cervical mucosa, endometrial fluid, immune cells, epithelial cells, and tissue exudates, which protects against pathogen entry, as well as aiding drug solubilisation in VDD [3,44]. In the vaginal wall, the common commensal Lactobacillus species, particularly *L. crispatus*, *L. gasseri*, *L. vaginalis,* and *L. jensenii*, cause the anaerobic breakdown of epithelial glycogen, resulting in lactic acid and hydrogen peroxide, which creates the acidic vaginal pH essential to maintain vaginal homeostasis. In addition, they also produce bacteriocin, which possesses antimicrobial activity while also impeding pathogen attachment to the vaginal mucosa, thereby creating a hostile environment for pathogens in the vagina [3,42,45,46,47,48]. Collectively, these factors contribute to the defence against cervico-vaginal infection and, typically, the presence of a vaginal infection is characterised by increased vaginal pH [42,49]. However, in the post-menopausal vaginal wall, there is a substantial reduction in glycogen and lactobacilli species driven by reduced oestrogen levels, increasing susceptibility to vaginal infection [35,42,49]. Fluctuating oestrogen levels are responsible for variation in vaginal fluid volume, microbiome content and diversity, and wall thickness over a woman’s life cycle, as shown in Table 2. For example, higher oestrogen levels encountered during reproductive age increase the metabolic activity of the epithelium, thereby adding cell layers and wall thickness. In contrast, atrophy leads to depleted wall thickness in the post-menopausal vagina [3].

Vaginal vasculature health directly correlates to the wall thickness and is maintained by branches from the inferior vesical artery and internal pudendal artery in the lamina propia and the plexus of Santorini, draining blood into the internal iliac vein, which avoids the hepatic first-pass effect. The muscular layer and tunica adventitia provide the elongating property of the vagina and supplements from the lymphatic system, respectively [3,43].

## 3. Vaginal Drug Delivery Systems: History and Present Therapeutics

Some of the earliest reports of administering medications directly to the vaginal cavity date back to 1850 BC, used primarily for contraception and treatment of vaginal inflammation. However, its clinical use was approved only in the late 1970s and significant advances have been made in the past five decades, particularly in the development of vaginal microbicides for HIV prophylaxis [19,50]. Given the advances in pharmaceutical technologies over the last two decades, preference has firmly moved towards the vaginal route of drug delivery for diseases/ailments of the FRT [18]. A variety of active pharmaceutical ingredients (APIs), including antibacterials, antifungals, antiprotozoals, anti-virals, labour inducers, spermicidal agents, and sexual hormones, have been formulated in vaginal dosage forms [4,20,51]. Nevertheless, vaginal use is restricted by cultural sensitivity, patient compliance, and the highly variable anatomical and physiological features of FRT over a lifetime, which needs to be borne in mind when designing products for vaginal delivery [50]. Moreover, the effect of a vaginal formulation on the adjacent healthy tissue should also be taken into account, since any damage could disrupt the vaginal microbiome balance and increase susceptibility to infection [19]. Therefore, it is crucial to consider all limitations, specifically physiological and anatomical factors, when designing vaginal products, so that correct administration ensures patient safety and optimal efficacy [19].

### 3.1. Drug Absorption from the Lower and Upper Female Reproductive Tract

As depicted in Figure 1, the FRT is categorised into lower and upper regions, with the stratified squamous epithelium of the vagina accounting for a major portion of the lower FRT [27]. Drug retention at the epithelial surface with minimal permeation and uniform distribution in the entire vaginal cavity is preferred for the local action of drugs [51,52]. In contrast, controlled and sustained drug absorption through the mucosal tissue is critical for the systemic effect of drugs delivered by the vaginal route. In this regard, drug permeation through the vaginal epithelial barrier occurs by the transcellular and/or paracellular pathway, depending on the physicochemical characteristics of the drug and how it is formulated [52]. Both of these routes of drug transport are preceded by two consecutive steps comprising initial drug dissolution in vaginal fluid followed by drug absorption/permeation across the epithelial membrane of the lumen, and they are affected by physiological and pharmaceutical parameters [51]. Transcellular absorption is driven by passive diffusion through epithelial cells, whereas paracellular diffusion occurs through the tight intracellular junctions, which are abundant in the endocervix but rare or absent in the ectocervix and vagina. These tight junctions serve as a line of defence, allowing the selective passage of fluid and small ions only [52]. Generally, small hydrophobic drugs are absorbed through the transcellular route, involving, in most cases, a simple diffusion process. In contrast, small hydrophilic drugs are most typically absorbed through the paracellular route, although the transcellular route is possible in the presence of larger molecules, or specific carriers/transporters [53,54].

Four mechanisms have been proposed for vaginal drug absorption and distribution to the uterus, which include (i) passive diffusion through the vaginal epithelium, (ii) passage of the drug through the cervical canal to the uterus, (iii) transportation from the utero-vaginal venous and lymphatic system, and (iv) counter-current exchange between utero-vaginal veins and/or the lymphatic system by diffusion [55,56].

Analogous to the hepatic first-pass effect, the uterine first-pass effect is observed due to concurrent exchange by diffusion through veins and arteries located in the upper third portion of the vagina and uterus, resulting in a substantial drug concentration increase in the uterus [52]. Preferential vagina-to-uterus distribution is suggested by several studies and results in significantly higher drug concentrations in the endometrium than in the blood circulation [57,58]. For example, the concentration of intravaginally administered ^99m^Tc-pertechnetate was found to be higher in vaginal spaces, lymph vessels, and the vaginal veins, indicating that drug distribution from vagina to uterus was primarily mediated by diffusion from the vagina, and a counter-current transfer mechanism [55]. In another study, where a poloxamer hydrogel infused with carboplatin and fluorescein sodium was administered intravaginally in mice, the dye disposition was observed at higher levels in the vaginal periphery; the result was the remission of cervical cancer, likely driven by higher drug levels in utero than the systemic circulation [59]. In a separate study designed to boost in vitro fertilisation, vardenafil given intravaginally exhibited remarkably higher maximal concentrations, C_max_ (32-fold), and area under curve, AUC_0–4h_ (20-fold), in the uterus compared to the uterine drug concentration following the administration of an oral vardenafil suspension [60]. Such transport kinetics are beneficial where a drug’s intended target is the uterus, particularly in the case of sex hormones [19].

#### 3.1.1. Physiological Factors Affecting Drug Absorption

As alluded to above, age, cyclical changes, pregnancy, coitus, and medications can induce FRT changes, which affect the vaginal microenvironment [7,61,62]. The impact of age and cyclical changes on vaginal and uterine physiology is summarised in Table 2. Such fluctuations in mucosal tissue characteristics have a direct impact on the absorption and efficacy of drugs delivered by the vaginal route [52]. A key factor dictating absorption is the degree of drug ionisation, with the unionised form primarily responsible for absorption and efficacy. Therefore, a fluctuating pH of the vaginal mucosa directly affects drug ionisation, and this in turn modulates drug absorption behaviour [63]. For instance, an elevated vaginal pH, which is present during yeast infection and sexual intercourse, can cause the ionisation of certain drugs, diminishing their absorption [64].

Likewise, an altered vaginal microbiome can lead to the colonisation of pathogenic bacteria on the epithelium of the vaginal mucosa [48]. This bacterial layer not only leads to vaginal infection, including bacterial vaginosis (BV), aerobic vaginitis (AV), HIV, HPV, and chlamydia, but also hinders drug absorption, as well as forming a barrier to prevent the local action of drugs, hence reducing clinical resolution [48,65]. Moreover, such biofilms also reduce the antimicrobial activity of some drugs [46]. For instance, in non-lactobacillus dominant vagina, tenofovir is metabolised by *G. vaginalis*, suggesting reduced anti-HIV efficacy [66].

Both pH and lactobacilli composition are directly correlated to oestrogen levels, which peak just before ovulation, when there is an abundance of lactobacilli or when the vaginal pH is relatively low [41,65]. In contrast, oestrogen levels fall to their lowest levels just prior to menstruation [65]. Additionally, oestrogen and glycogen levels fall when approaching menopause, resulting in marked reductions in vaginal glycogen content and lactobacilli populations, which drives up the vaginal pH as glycogen and lactobacilli are major prerequisites of lactic acid production [67,68]. Such dynamics cause varied absorption profiles in different population groups. In another study using tenofovir, higher rates of efficacy were observed in patients whose vaginal microbiome predominantly comprised lactobacilli [46]. Since lactobacilli are in abundance in females of reproductive age, this suggests that a reduced efficacy of tenofovir can be expected during HIV prevention in post-menopausal women [40,46]. Moreover, the vaginal epithelium thickness also changes with age, as well as in pregnancy and cyclical stages, and while drugs are readily absorbed through the vaginal route before puberty, absorption decreases as females reach puberty due to thickening of the vaginal epithelium. For instance, penicillin absorption diminishes during the follicular phase of menstruation, and a similar trend is observed in the last trimester of pregnancy [69].

Interestingly, unlike the other mucosal drug delivery routes, uterine drug absorption does not depend upon the ionisation of a drug. Rather, absorption is driven by the charge of the drug, making the uterus selectively permeable to drugs that are negatively charged or possess a lower overall net charge, while also being readily permeable to high-molecular-weight (MW) drugs [70].

Enzymatic activity within the FRT is another physiological factor affecting drug stability and bioavailability, despite it having much lower enzymatic activity than the gastrointestinal tract [3]. The reduced bioavailability of proteins and peptides, for example, is attributed to aminopeptidase enzymes prevalent in the vaginal mucosa [49].

Likewise, both the volume and viscosity of vaginal fluid are important parameters that affect drug absorption, with ovulation triggering an increase in mucus volume and reduced viscosity [48]. The change in volume and viscosity of vaginal fluid at different stages of females’ life cycle is presented in Table 2. Vaginal fluid can lead to the dilution and/or rapid clearance of vaginal formulations, which results in alterations of the rheological and mucoadhesive properties of the drug formulation, ultimately affecting the drug’s residence time in the FRT [71]. As expected, highly viscous mucus acts as a barrier to drug absorption, while a less viscous and higher volume of mucus would clear the drug prematurely, reducing its absorption and efficacy [39]. Nevertheless, sufficient fluid volume (0.5–0.75 mL) is required if the drug is not in solubilised form, while increased fluid volume could also augment the absorption of poorly water-soluble drugs [49,69]. Therefore, consideration of these aspects is of paramount importance while designing a drug formulation, so as to ensure that it is conducive to optimal drug retention and absorption.

Considering the strong impact of vaginal fluid on various attributes of the formulations, such as pH, osmolality, rheology, and mucoadhesion, characterisation of vaginal formulations is done in simulated vaginal fluid (SVF) [72]. SVF has pH, osmolality, and viscosity identical to the vaginal fluid; hence, investigation of the technological aspects of vaginal formulations using SVF is beneficial to predict the in vivo performance of such formulations [44].

#### 3.1.2. Physicochemical Properties of Drug and Excipients Affecting Absorption

Drugs having optimal physicochemical properties can be further exploited to achieve the maximum therapeutic outcome through appropriate formulation design and drug delivery strategies [73]. Xenobiotics coming into contact with the FRT mucosa are typically entrapped and cleared along with ciliated mucosa, while those possessing favourable characteristics, such as low MW, optimal charge/pKa, solubility, and hydrophobicity, can circumvent vaginal entrapment and clearance, and hence experience greater rates of mucosal absorption [74,75]. However, these desirable physicochemical attributes can be stymied by the dynamic changes of the FRT; therefore, thorough assessment and consideration of these physiological features is needed when designing sophisticated formulations [76].

Weakly acidic drugs (pKa < 5.5, e.g., tenofovir) remain unionised under physiological conditions, while weakly basic drugs (pKa 8.5–10.5, e.g., itraconazole, voriconazole) are ionised in the acidic environment of the lower FRT [77,78,79,80]. However, a fluctuating pH, especially that evident in the vagina, can directly affect drug ionisation, which in turn impacts solubility and ultimately drug absorption. Solubility can also be enhanced by reducing the drug’s MW. Smaller-sized lipophilic drugs (e.g., itraconazole, efavirenz) and hydrophilic agents (e.g., mannitol) are preferably absorbed through the tight junctions in-between epithelial cells [49,52,81,82]. 

Likewise, the physicochemical nature of a drug also determines the pathway of drug diffusion. Bradykinin, being hydrophilic, diffuses through the paracellular pathway and its diffusion across the vaginal epithelial cells is retarded due to the interaction between the negatively charged epithelium and two positively charged arginine groups in bradykinin, suggesting that the drug–drug molecule charge is also a factor impacting drug absorption [83]. This could indeed be a basis to discriminate various molecules for desired local versus systemic effects [5]. Nevertheless, vaginally administered drugs should overcome both mucosal and epithelial barriers for effective absorption. While hydrophilicity is essential for mucosal drug absorption, hydrophobicity facilitates epithelial cell internalisation. Ideally, for dosage forms such as tablets and gels, an optimal hydrophilic–lipophilic balance is sought for maximal absorption, which can be modulated through appropriate excipient selection and dosage form design [74,84], making Log *p* more critical than MW for the success of such formulations [84]. In the case of vaginal rings, however, particle size is more crucial due to the hydrophobic polymers used in their preparation, where the drug must initially dissolve in the polymeric matrix and then diffuse through the matrix, and this then ultimately needs to dissolve in the aqueous vaginal fluid. Generally, drugs with MW less than 1000 Da and a Log *p* value in the 2–4 range are desirable for vaginal rings [84]. An interesting case in point is where the vaginal distribution and retention of doxorubicin (DXN) was increased by formulating hypotonic mucoinert nanoparticles. Here, the hypotonicity resulted in epithelial cells absorbing extracellular fluid to help revert to an isotonic state, and this influx of water carried DXN, alongside water, into cells [83,85]. These examples illustrate the value and important role that excipients play in attaining optimal drug absorption and retention, an aspect that is extensively explored in later sections.

### 3.2. Gynaecological Conditions and Their Management Using Conventional Dosage Forms

Medical interventions are necessary across the various phases of the female reproductive life cycle, which can require pharmacotherapy in the case of gynaecological diseases (Figure 1), pregnancy, childbirth, and post-menopause [15,86,87,88]. In this regard, and with a focus on VDD, there are a suite of conventional dosage forms with their inherent advantages and disadvantages, which are highlighted in Table 3 below.

**Table 3:** Key features and attributes of commercial vaginal dosage forms.

Dosage Form	Formulation Features	Advantages	Disadvantages	Active Agents	References
Insert/ Tablet/ Capsule/ Pessary	Rod/conical/wedge- shaped, disintegrates/dissolves, releasing drug locally in the vaginal cavity	Ease of administration and retrieval when use is undesirable, fast/slow dissolving, user-friendly, increased drug stability in tablet and capsule formulation, economical	Vaginal leakage, reduced drug residence time, diminished drug stability in pessaries	Oestrogen, Dinoprostone, Clotrimazole mucoadhesive tablets, Lactobacillus plantarum capsule	[37,86]
Gel	Formed by chemical bonding or physical entanglement between the polymeric chains	Ease of application, adequate spreading across the vaginal mucosa, and enhanced patient comfort	Poor drug retention	Metronidazole, Dinoprostone	[3,6]
Ointment	Drug dissolved in aqueous phase and mixed in oil phase	High acceptability, easy administration	Leakage, multiple administrations required to attain maximum therapeutic benefit	Terameprocol	[14,72,89]
Sponge	Solid porous structure with dispersed gas in solid matrix	Ability to load higher drug amount, drug released under the pressure exerted by movements of FRT	Mucosal irritation	Nonoxynol-9	[3,90]
Cream	Biphasic system, dissolved in internal phase and dispersed in external phase	High acceptability, prolonged vaginal drug residence time	Leakage, multiple administrations required to attain maximum therapeutic benefit	Clindamycin	[14,49,72,91,92]
Cervical patch	Bilaminar sheet of bioadhesive layer containing drug and backing layer	Reduced drug exposure to surrounding vaginal tissue	Limited rate of drug load	5-fluorouracil	[59]
Vaginal ring	Circular devices with controlled drug release pattern where initial burst release is followed by steady-state drug release	Controlled and sustained drug release profile, reduced exposure of drug to adjacent tissue, no leakage problems, economical, applicator not required, better patient compliance	Uncomfortable, limited rate of drug load and drug molecules, higher drug waste, irregular drug distribution	Clotrimazole, dapivirine	[14,37,49,59,93,94]
Vaginal film	Fast/slow-dissolving polymeric film, which dissolves on vaginal mucosa	Good drug retention in vagina, fast/sustained release, no disturbance to normal vaginal microbiome, not messy, compatible with various drugs, better stability of drug	Inconvenience of administration	Dapivirine, itraconazole	[14,95,96]

- Vaginal infections

Vaginal infections, which include BV, AV, and vulvovaginal candidiasis (VVC), are the most common causes of women seeking medical consultation and pharmacotherapy [97]. They typically manifest from a disruption to the fragile vaginal microbiome, which can be triggered by stress, medications, or coitus [97,98]. For example, a decline in lactobacilli species and overgrowth of anaerobic bacteria, particularly *Gardnerella vaginalis, Prevotella*, and *Mobiluncus* species, are observed in BV [98]. Although not directly related to increased mortality, an infection, particularly when it becomes chronic, can severely affect mental health, self-confidence, sexual relationships, and overall quality of life [86,97]. They also increase the susceptibility to contracting sexually transmitted infections (STIs), including gonorrhoea, chlamydia, HPV, and HIV, which can lead to infertility, secondary complications in pregnancy, as well as pelvic inflammatory disease (PID), cervical cancer, and endometriosis [91,98].

Numerous treatment options are available commercially in a host of conventional dosage forms, as shown in Table 4, for the management of vaginal infections. However, they too suffer from several drawbacks (presented in Table 3), limiting the therapeutic outcomes. Moreover, increasing concerns of antimicrobial resistance and recurrence of BV still remain unacceptably high, which could in part be due to the inability of drugs to effectively penetrate/compromise pathogenic biofilms [97,98,99]. Hence, prolonged therapy might be required to prevent reoccurrence, with complicated cases requiring even intense, prolonged, and multidrug regimens through both oral and topical routes [97,100]. Therefore, formulations engineered to perfuse and disrupt biofilms are sought, as they are expected to support the complete resolution of vaginal infections, while preventing recurrence. Although novel formulation strategies have led to significant improvements in reducing the dosage regimen, it was still a long time before their clinical application was defined [97]. This warrants the rational design of smarter, robust formulation strategies that align with disease pathology, which can be exploited by appropriately engineered sol–gel formulations, as is explored in later sections.

- Cervical ripening, labour induction, and childbirth

Despite the natural phenomenon of cervical softening, thinning, and dilation during childbirth, 25% of pregnancies require medical induction procedures that utilise prostaglandins [101,102]. Misoprostol is available for vaginal, oral, buccal, and sublingual administration. However, the vaginal administration of misoprostol exhibits rapid delivery and requires lower doses compared to buccal administration [101]. Vaginal administration was also reported to have higher efficacy than the buccal or sublingual routes in two separate studies [103]. In contrast, no significant differences in delivery time between vaginal and buccal administration of misoprostol were observed in a separate study [15]. Despite these contradicting findings, vaginal delivery still has superseding therapeutic benefits, such as the rapid induction of cervical ripening and low dose requirement [15,103]. Furthermore, among the vaginally administered tablets, inserts, and gels of dinoprostone, tablets were found to be most effective; however, a low rate of patient acceptance constrains its broader use [104]. Further research is therefore needed in the design of vaginal prostaglandin formulations, where higher efficacy and patient acceptance can be attained.

- Prophylaxis of HIV

According to the United Nations Program on HIV and AIDS (UNAIDS) 2020, 50% of the newly diagnosed HIV cases are represented by females, with sexual intercourse being the predominant route of transmission [105]. Semen not only acts as a carrier of HIV, but also fuels vaginal pH neutralisation, which supports virus attachment to the cervicovaginal mucosa [14]. The virus then infects the CD4+ and CD8+ T and dendritic cells in the vaginal wall initially; this attracts the release of cytokines, which expand in number locally, ultimately reaching the systemic circulation. This cascade overrides the vaginal defence mechanism responsible for protecting against virus invasion, making sexual intercourse the leading means of HIV transmission [14,106].

Based on the mechanism of virus invasion, vaginal microbicides were developed to have adequate epithelial permeability and acceptable pharmacokinetic and safety profiles, as well as targeting the critical steps of HIV transmission [14,107]. In addition, changes in vaginal pH and the dilution of microbicides are also considerations not to be overlooked, since seminal fluid can collectively lead to dilution, three units of change in pH, and increase drug interaction with fructose moieties present in semen [108].

Unfortunately, drugs or vaccines with desired characteristics that reliably prevent HIV transmission are not available yet and, therefore, the use of already established microbicides continues [107]. Microbicides including terameprocol, nonoxyl-9, dapivirine, tenofovir, and lersivirine are in various stages of development for vaginal use in the prevention of HIV transmission [14,109]. However, considering the drawbacks of the conventional vaginal gels and rings, a more promising dosage form that can impart on-demand drug release in a controlled manner would be a great advancement [106]. This requirement could be achieved by engineering drug formulations with highly controlled rheological properties. Hence, the exploration of novel strategies is deemed essential for the prophylaxis of HIV in the vulnerable demographics and where exploitation is rife.

- Atrophic vaginitis (AV)

Declining levels of oestrogen accompanied by atrophy of the vagina are encountered when approaching menopause, with symptoms including vaginal dryness, irritation, and dyspareunia having an impact on sex life, relationships, and overall quality of life [88]. Various hormonal (use of oestradiol and oestratriol) as well as non-hormonal (use of lubricants, moisturisers, and probiotics from isolates of healthy vagina and uterus) strategies are employed to enhance patient comfort in these cases. However, their delivery using conventional formulations is associated with considerable discomfort, and adverse effects that lower user adoption, warranting the development of novel drug formulations that directly address and mitigate these shortfalls [88,110].

- Pelvic inflammatory disease (PID)

PID is a common infection of the upper FRT, and is most prevalent during the reproductive years. The overgrowth of pathogenic microbiomes disturbs the vaginal and cervical defence mechanisms and they ascend towards the upper FRT, resulting in dysbiosis and infection [111]. If left untreated, PID may be associated with chronic pelvic pain, ectopic pregnancy, infertility, endometriosis, and ovarian cancer [111,112,113].

Oral combinations of doxycycline and metronidazole are widely used in the treatment of PID but are associated with several unwanted effects, resulting in reduced adherence to treatment. In contrast, localised delivery provides the potential for sustained drug release and enhanced antibacterial activity [114]. Development of novel formulations that have the capacity to deliver drugs more efficiently to the deeper areas of the upper FRT could provide a better, more complete, and curative option for PID.

- Endometriosis

Endometriosis is the debilitating condition of the outgrowth of the uterine epithelial and stromal tissue beyond the uterine cavity, characterised by dysmenorrhea, dyspareunia, chronic pain, and infertility [111,115]. Even though it is caused by high oestrogen receptor expression, a retrospective study demonstrates a higher risk of endometriosis among females with PID [16,111].

Treatment of endometriosis uses various hormonal drugs, including progestins, and GnRH analogues and antagonists, aromatase inhibitors, oestrogen and progesterone receptor modulators, as well as non-steroidal anti-inflammatory agents, delivered via a variety of routes [16,115]. However, the uterine first-pass effect opens the way for VDDS to be a highly promising approach for the management of endometriosis [116,117]. For instance, the intravaginal administration of danazol ameliorates pain in endometriosis with less androgenic side effects [118]. Likewise, the steroid hormones oestrogen and progesterone are used intravaginally for endometriosis management [56,116]. However, considering that the uterine first-pass effect is localised to the upper third region of the vagina and counter-current exchange between vagina and uterus occurs in this region, targeted vaginal administration is considered crucial [84,119]. In fact, suboptimal therapeutic effects have been reported when steroids are placed at an inappropriate depth in the vagina [120]. Therefore, designing formulations and delivery applicators/devices that deliver and retain formulations for longer periods in the upper third portion of the vagina could augment the therapeutic benefits of agents indicated for endometriosis.

- Contraceptives

Hormonal contraceptives based on combined oestrogen and progestin, or progestin alone, are used widely for contraception as well as non-contraceptive purposes such as irregular periods, heavy bleeding, and anaemia, and are available as combined oral contraceptives (COCs), emergency pills, intramuscular depots, subdermal implants, and intrauterine devices [120,121]. Despite the availability of several hormonal vaginal and non-vaginal contraceptives, vaginal contraceptives are gaining in popularity due to the advantages that the vaginal route offers compared to non-vaginal routes [78]. Nevertheless, high costs, side effects such as weight gain, low libido, depression, increased risk of breast cancer, cervical cancer, and venous thromboembolism, the necessity of strict adherence to the dosage regimen of COCs, and the requirement of trained personnel for vaginal use reduce patient compliance [78,121,122]. This has resulted in almost 99 million unplanned pregnancies worldwide annually, highlighting the need for novel controlled-release contraceptives that can reduce side effects, and so aid in patient compliance and adherence [78,121,122]. Recently, in May, 2020, a relatively safer and more convenient non-hormonal vaginal contraceptive gel (Phexxi^TM^) was approved by the U.S. Food and Drug Administration (FDA), which is appealing to women not preferring hormonal drugs due to pre-existing medical conditions or simply wanting to avoid hormone-associated side effects [123].

- Infertility

Female infertility can arise from a number of conditions, including endometriosis, adenomyosis, uterus fibroids, congenital uterine anomalies, polycystic ovarian syndrome (PCOS), thyroid disease, fallopian tube blockage, PID, and cervical problems, resulting from an inappropriate quantity/quality of cervical mucus [23,26]. It is treated by the replacement of oestrogen and progesterone hormones, ovulation induction or superovulation using FSH and LH, or assisted reproductive technology, which needs recombinant human FSH or urinary gonadotropin hormones [124,125]. However, the oral administration of hormones is associated with an increased risk of heart disease, pulmonary embolism, stroke, and breast cancer [87,88]. Furthermore, low doses of GnRH agonists or oral contraceptives used prior to in vitro fertilisation in endometriosis-related infertility often fail to address the systemic effects of the therapy [115]. On the other hand, higher doses of hormones increase their efficacy but result in severe side effects, such as weight gain, uterine bleeding, mood changes, and even intolerance [78,115]. In such circumstances, VDD can safely deliver these hormones to the target site with minimal systemic effects and improve the patient experience and fertility rate.

- Polycystic ovarian syndrome (PCOS) and ovarian cancer (OC)

A disorder of the endocrine system characterised by diminished FSH and elevated LH levels can lead to ovarian cysts, and ultimately to ovarian cancer [126]. Apart from lifestyle and dietary modification, oral anti-diabetic drugs, particularly metformin, are used in PCOS because of the increased occurrence of type 2 diabetes, owing to the emergence of insulin resistance, which is commonplace in PCOS [127,128]. However, similar results offered by the vaginal administration of metformin niosome-infused thermoresponsive gels, allowing lower doses and remarkably few side effects, have paved the way for the development of vaginal dosage forms of metformin [126]. Moreover, oral and vaginal adminstration of micronised progesterone has also shown progesterone and LH levels similar to the mid-luteal phase, which is essential in PCOS management [129]. The results suggest that drugs can be customised, with tailored formulations to enhance drug delivery through the vaginal route for these debilitating conditions.

- Cervical cancer

Cervical cancer is the most common cancer in women and is characterised by abnormal epithelial tissue in the cervix, resulting primarily from HPV infection [59]. Treatment strategies for cervical cancer depend upon the stage of cancer as well as the necessity for the patient to carry future pregnancies. It is estimated that almost 80% of cervical cancers are diagnosed at a local stage and hence localised drug delivery, which minimises side effects, is highly desirable [59,130]. In fact, the vagina permits the easy localised delivery of a range of commonly used chemotherapeutic agents, including paclitaxel, cisplatin, topotecan, nonoxynol-9, 5-fluorouracil, docetaxel, oestrogen, mitomycin, disulfiram, and doxorubicin, which are used in the early stages of cervical cancer, and post-surgery to prevent reoccurrence [10,59,84]. Furthermore, the complete tumour suppression achieved by the intravaginal administration of cisplatin compared to systemic administration forms a basis for the future localised vaginal delivery of other highly potent chemotherapeutics [130]. However, the greatest challenge in the vaginal use of anticancer drugs is to have control over the drug release rate and drug distribution to minimise side effects. Moreover, the high probability of drug disposition beyond the cervix and to the uterus due to the large vaginal surface area and intact uterine first-pass effect needs to be considered and mitigated [59]. Advanced drug formulation designs that can provide controlled drug release characteristics, with minimal exposure to adjacent tissue, are highly desired for the localised management of cervical cancer (Table 4).

**Table 4:** Marketed VDD formulations, their indication, and the manufacturer.

Active Drug	Brand Name^®^	Dosage Form	Indication	Manufacturer	Reference
Oestradiol	Vagifem	Tablet	Atrophic vaginitis	Novo Nordisk Health Care AG	[131]
Dinoprostone	Prostin E_2_,	Tablet	Cervical ripening and labour induction	Pfizer	[104]
Dinoprostone	Cervidil	Insert	Cervical ripening and labour induction	Forest Laboratories	[49]
Misoprostol	Misodel	Insert	Labour induction	Ferring Pharmaceuticals	[132]
Progesterone	Endometrin	Insert	Assists embryo transplantation	Ferring Pharmaceuticals	[133]
Oestradiol	Imvexxy	Inserts	Atrophic vagina	Therapeutics MD	[88]
Clotrimazole	Gino-Canesten	Cream	Vulovaginal candidiasis	Bayer	[72]
Sertaconazole	Sertopic	Cream	Vulovaginal candidiasis	CPH	[72]
Clindamycin	Dalacin V	Cream	Antibacterial	Pfizer	[72]
Z. multiflora	Leucorex	Cream	Trichomoniasis	Barijessence	[134]
Oestriol	Ovestin	Cream	Oestrogen hormone supplement	Aspen	[72]
Etonogestrel/ Ethinyloestradiol	Nuvaring	Ring	Endometriosis, cervical cancer	Organon	[3,24,59,94]
Progesterone	Progering	Ring	Release progesterone	Laboratorios Andrómaco	[94]
Oestradiol	Estring	Ring	Oestrogen replacement therapy, cervical cancer	Pfizer	[3,59]
Nonoxyl-9	Today	Sponge	Spermicide	Almatica Pharma, Inc.	[3]
Progesterone	Crinone	Gel	Assisted reproductive procedures	Merck	[135]
Nonoxynol-9	Vaginal Contraceptive Film	Film	Spermicide	Apothecus	[3]
Lactobacilli gasser and Lactobacilli rhamnosus	EcoVag	Capsule	Bacterial vaginosis	HÄLSA Pharma GmbH	[45]
Progesterone	Utrogestran	Capsule	Luteal phase support	Laboratories Besins International	[136]

### 3.3. Current and Emerging Trends in the Treatment of Gynaecological Conditions

The direct vaginal administration of drugs to treat localised/systemic conditions of the FRT has significant benefits over oral administration for various therapeutic agents, including antimicrobial agents, contraceptives, labour inducers, and spermicides [11,50,97]. Most marketed conventional vaginal dosage forms to treat self-limiting conditions are self-administered by patients. However, these dosage forms present multiple limitations, including the unpredictable coating over the vaginal mucosa, and low drug penetration to the submucosal layers, which is compounded by premature leakage [2]. As a result, many conventional vaginal dosage forms fail to achieve their therapeutic potential, which warrants pharmaceutical scientists to reimagine treatment modalities for acute and chronic gynaecological conditions.

Nonetheless, notable breakthroughs have come from the availability and inclusion of versatile polymers within pharmaceuticals to facilitate formulation residence time at target site and the transport of drug molecules into the mucosal tissue, thus improving bioavailability; these have been adopted for vaginal solid/semi-solid dosage forms, films, and rings, imparting controlled and sustained drug release properties [37]. For instance, tenofovir has demonstrated extended vaginal drug residence and release times, of 96 and 72 h, respectively, by the use of hydrophilic polymers with mucoadhesive characteristics, such as hydroxypropyl methyl cellulose (HPMC) and chitosan [137]. The beneficial effects of using mucoadhesive polymers in localised drug delivery have been extended to hormones, spermicides, and antibiotics, while the systemic effect of contraceptives has been enhanced through extended drug retention. From a patient perspective, such polymers have also assisted in addressing the practical challenges of semi-solid dosage forms, such as leakage [37]. Mucoadhesive polymers used in soft gelatin capsules of oestradiol provided flexibility of drug positioning, faster dissolution, and higher satisfaction among 85% women, compared to women using conventional vaginal inserts and cream formulations of oestradiol [138].

Polymer technology has also evolved, with electrospun fibre technology being used to regulate the vaginal drug release of anti-HIV drugs and contraceptives [139]. Such platforms have the potential to deliver diverse classes of drugs, including small molecules, nucleosides, proteins, antibodies, and peptides, as a single drug entity or multiple drugs, as well as offering the encapsulation of drugs of varied solubility, which can assist in filling the considerable voids that exist with conventional dosage forms [139,140,141].

Furthermore, polymers are also used to entrap bioactive agents in a technique known as encapsulation, resulting in the formation of micro- and nanoparticles, which aim to improve drug stability and facilitate controlled and targeted VDD. Various methods of drug encapsulation, including spray drying, inclusion complexation, emulsification, freeze drying, coacervation, liposome preparation, etc., are employed [142,143,144]. However, the conventional encapsulation methods have their own merits and challenges. For instance, although spray drying is an easy encapsulation method and can be scaled up for industrial use, it is not suitable for heat-labile drugs as it uses a high operational temperature, which can lead to drug degradation and lower drug stability. Therefore, novel encapsulation techniques devoid of harsh conditions of temperature, pressure, and chemicals are receiving increasing attention [142]. This includes the use of novel micro- and nano-sized systems [142]. Both microparticles (5–300 µm diameter) and nanoparticles (1–1000 nm diameter) are technically similar as they are prepared under comparable conditions [145,146]. Microencapsulation offers a solution to the issues of multiple or frequent drug administration, higher stability, controlled and sustained drug release, as well as the delivery of hydrophilic and hydrophobic drugs [145,147]. However, maintaining the bioactivity of a drug is highly challenging in microencapsulated formulations, which could be possibly solved by the utilisation of emerging technologies [145]. For instance, the electrospraying microencapsulation technique has been shown to generate better outcomes than conventional formulation methods. Microencapsulation of dry extracts of various electrosprayed phytoformulations has improved solubility and vaginal bioavailability [148]. Likewise, an increase in the survival rate of probiotics was reported by using this technique, indicating their promise in VDDS [149].

Similarly, nano-sized systems, which include nano-emulsions, vesicles, particles, liposomes, dendrimers, and cyclodextrin, also have an array of benefits over conventional formulations [150,151]. Such systems not only shield the impact of physiological factors on drugs, but also exploit such factors for controlled drug delivery, such as the utilisation of pH for pH-stimuli-responsive drug release [151]. They facilitate drug distribution over the vaginal mucosa through mucoadhesion, thus preventing rapid drug clearance and greater permeation to the underlying mucosal tissue, which are of paramount importance in overcoming the physiological resistance imparted by the complex cervicovaginal mucosal layer [19,151]. In addition, regardless of their physicochemical nature, the diverse nature of drugs can be encapsulated in nano-systems for delivery by the vaginal route, which also serves to protect drugs from enzymatic and hydrolytic degradation, and modulating the cervicovaginal pH and fluid volume leads to enhanced drug stability [150]. Although both nano- and microencapsulation have their own advantages, nanoparticles in particular have a greater capacity to enhance drug solubility, dissolution, lipophilicity, permeability, and stability, which is particularly advantageous for poorly water-soluble drugs. Likewise, the higher surface-to-volume ratio of nanoparticles makes them more effective for targeted drug delivery [146,152]. Such features of polymeric nanotechnology have resulted in enormous attraction in the development of vaginal microbicides in the past decade [152]. Additionally, the relatively safe and easy preparation of nanoparticles due to the use of non-aseptic initial preparation conditions and less toxic solvents, as well as the higher drug load owing to higher adsorption in a larger surface area compared to microparticles, provides added benefit [153]. However, both micro- and nanoparticles require an efficient vehicle for delivery to the FRT mucosa. In light of the array of benefits that nanoparticulate systems bring, their incorporation into a suitable vehicle warrants investigation, and, as elaborated below, the sol–gel platform has gained much attention, with some promising prospects in the context of VDD [150,154].

The importance of mucosal drug delivery, based on the principles of mucoadhesion and mucopenetration, cannot be overstated in VDD, as they form the basis of controlled and extended pharmacokinetics. A formulation should ideally adhere to the mucosal layer of the vagina, which has a low rate of mucosal turnover at ≈1.5 mL/day, and a flow rate of 6 mL/day. Drugs incorporated into mucopenetrating systems exhibit better spreadability and penetration into deeper layers of the vaginal epithelium, rendering them suitable for both localised as well as systemic therapy [155]. Mucus-penetrating particles (MPPs) of chemotherapeutic agents have substantially improved the quality of life of patients during and after treatment in the early stages of cervical cancer [84]. Paclitaxel-loaded MPPs suppressed tumour growth more effectively and doubled the survival rate of mice compared to unencapsulated paclitaxel and conventional nanoparticles of paclitaxel, suggesting that paclitaxel-loaded MPPs better infiltrate tumour cells over conventional nanoparticles [156]. Additionally, sol–gel formulations have drawn considerable attention in the scientific community as they have been proven as efficient vehicles for the local and systemic delivery of an array of drugs with varied physicochemical properties, including nano-/microparticular systems, which are explored in detail below [1].

## 4. Sol–Gel Platform Technology in Vaginal Drug Delivery System

**Figure 2:**
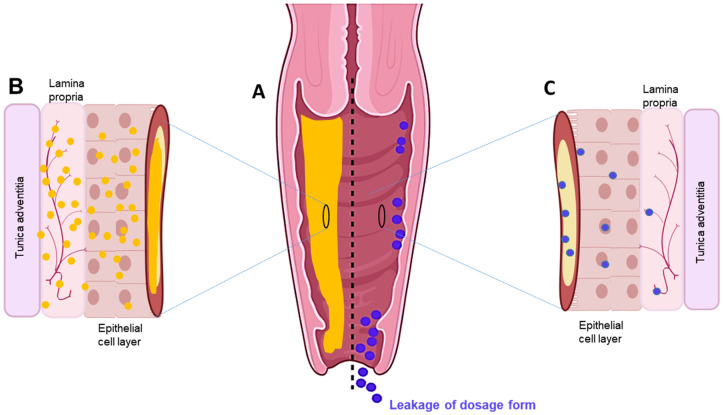
(**A**) Cross-section of the vaginal tract. (**B**) Uniform distribution and diffusion of drug throughout mucosal–epithelial layer with sustained delivery using an in situ sol–gel system. (**C**) Poor and sparse drug distribution through mucosal-epithelial layer and leakage via conventional dosage forms.

The vagina is an appropriate site for local and systemic drug delivery to treat/manage a broad range of gynaecological conditions [50]. A range of intravaginal medications are available on the market, with most requiring frequent application/administration due to the associated key limitations of short residence time and inadequate drug distribution on/through the vaginal mucosa (Figure 2) [3,4]. In this context, there is a case to be made for sol–gels, which can be engineered to prolong the vaginal residence time, resulting in predictable drug disposition to the vaginal mucosa (Figure 2) [4,97]. Sol–gels, also referred to as in situ hydrogels, which involve phase transition simply in water, have shown promising results in terms of protein and peptide drugs, tissue engineering, and overcoming barriers to drug absorption [157]. With their combined attributes of both solutions and gels, they support polymer-induced drug solubilisation, and uniform drug distribution, when applied to the vaginal mucosa with an appropriate applicator/device [11]. Once in the gelled state, increased viscosity and incorporation of excipients that promote mucoadhesion reduce vaginal outflow/leakage and mitigate the loss of the drug from the vaginal cavity, providing greater opportunity for drug absorption in/through the vaginal mucosa and favourable drug release kinetics [31,158]. Thus, overall, an appropriately engineered mucoadhesive in situ sol–gel system can bring a multitude of benefits that support the attainment of desired clinical outcomes in the FRT.

### 4.1. Features and Use of Vaginal Sol–Gel Formulations

The development of a drug delivery system using stimuli-reactive smart polymers, as is the case with sol–gels, allows fine-tuning of the rheomechanical properties aligned to the use, be it localised (deposition) or systemic (permeation) drug delivery that is sought [158,159]. These smart polymers tend to transform their characteristics in response to physiological changes, with the extent/degree of transformation determined by the nature of the monomer(s), charge density, pendant chains, and the degree of polymer crosslinking [160]. Stimuli-responsive in situ sol–gel systems are a unique dosage form, being a clear, low-viscosity polymeric liquid/solution, which, through a given trigger (pH, temperature, ions), converts to a viscous gel upon administration into a body cavity [158]. From a thermodynamic viewpoint, the balance between the hydrophobic and hydrophilic groups on the polymer chain and the free energy of mixing (ΔG=ΔH−TΔS) result in marked alteration of the aqueous solubility of the polymer and cause sol-to-gel phase transition [1,6]. Since the enthalpy (ΔH) is smaller than the entropy (ΔS), an increase in temperature (T) results in negative free energy of association (−ΔG), which increases the preference of polymer-polymer and water-water interactions to polymer–water interactions and causes the dehydration of solvated polymers [1,161]. In the case of amphiphilic polymers, an increase in polymer concentration above CMC results in the packaging of micelles in an ordered manner, forming a hydrophobic core and hydrophilic shell, and ultimately forms a gel [1,161,162]. Various mechanisms of stimuli-responsive gelation are shown in Table 4.

The stimuli-responsive polymers exhibit pseudo-plastic behaviour, which is highly desirable to formulate sol–gels as they offer better distribution on the mucosal tissue [162]. For the drug formulation exhibiting such behaviour, viscosity is reduced during application on the mucosa due to the change in alignment of tangled polymeric chains in the shear direction. However, the original rheological profile is regained immediately after application on the vaginal mucosa in response to the physiological stimuli [6,162]. This leads to the formation of a viscous gel in the vaginal cavity, which is essential for prolonged drug residence time in the vaginal cavity and ultimately results in sustained and controlled drug release [158]. The sustained and controlled drug release profiles exhibited in the examples of sol–gel systems included in Table 4 are attributed to the increased vaginal residence time of the drug formulations. Further, drug distribution in the mucosal tissue is also assisted by the ability of the gel to permeate the mucosa [6]. For instance, a significant increase in drug permeation was observed with a vaginal in situ gel formulation of fluconazole, which could be attributed to both the improved drug release profile of the drug in the formulation as well as the interaction between chitosan used in the formulation and mucin present in the vaginal mucosal layer [163]. Studies on VDDS report an increase in drug permeability by the use of polyethylene glycol [164].

**Figure 3:**
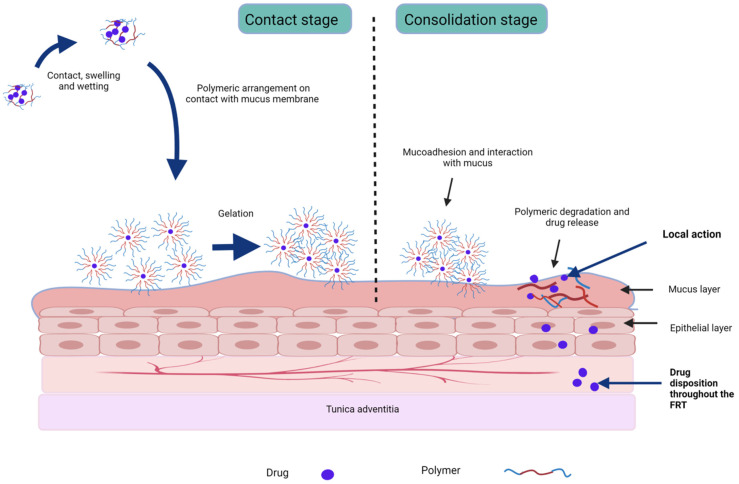
Stages of mucoadhesion and drug release from stimuli-responsive sol–gel formulations.

Mucoadhesive in situ gelling systems are those (containing natural or synthetic mucoadhesive polymers) that interact with and adhere to the mucosal epithelial surface components, particularly mucin, via hydrogen bonding, electrostatic interaction, and van der Waals forces, and, once administered in soluble form, they rapidly undergo in situ gelation (Figure 3) [8,47,50]. Published results suggest that at least 6 h of vaginal drug residence time is desired to represent the mucosal clearance turnover rate for the drug to cross the mucosal barrier (30–100 µm thick), epithelium layer (200–300 µm), and other adjacent layers before reaching the blood vessels in the vagina. This target can be met by a gel using mucoadhesive polymers containing suitable surface characteristics, charge, and functional groups, such as hydroxyl, sulphate, carboxyl, and amine groups—for example, polymers such as polyacrylic acid, cellulose, chitosan, hyaluronic acid, carrageenan, alginate, gums, and sulphated polysaccharides [6,7,31,50]. Use of these polymers results in optimum mucoadhesive strength, sustained drug release, and increased drug uptake by the vaginal mucosa [7]. Mucoadhesive systems not only improve bioavailability through localised action but also alter tissue permeability and enhance the absorption of protein and peptide-based drugs [165]. This makes mucoadhesion an essential parameter to consider and optimise as per the requirements to obtain formulations for controlled and sustained deliveries [20,166]. For instance, in a study comprising a thermosensitive formulation, mucoadhesive formulation, and thermosensitive–mucoadhesive system, the in situ mucoadhesive gel was considered the optimum formulation, with a longer vaginal residence time compared to the two other systems (>8 h). The study suggests that both gelation and mucoadhesiveness together result in a robust vaginal formulation [164].

Mucoadhesion occurs in two stages: (i) contact stage involving hydration, wetting, and spreading; (ii) consolidation stage involving strengthening polymer and mucin interactions through hydrogen bonds, hydrophobic interactions, van der Waals forces, electrostatic interactions driven by negatively charged mucin, and/or mucoadhesive or polymer chain interpenetration into the cervicovaginal mucus gel (Figure 3) [6,7,166]. Mucoadhesive intravaginal formulations should be engineered considering the nature and physico-chemical characteristics of drugs and their transportation route to overcome the associated challenges and improve their therapeutic efficacy [167]. In addition, for vaginal application, these polymers should be non-toxic, non-irritating, flexible, comfortable, and ideally remain unabsorbed in the vaginal epithelium [37]. The various polymers used in VDDS are represented. Natural polymers used in these systems typically respond to single or multiple stimuli, while synthetic polymers respond to specific stimuli. However, a major issue with the use of synthetic polymers is that they can result in irritation and toxicity to the underlying tissue [158]. Hence, diligent polymer and excipient selection is necessary before formulation into sol–gel systems, for either localised or systemic action.

In this context, the World Health Organization (WHO) has issued guidance for vaginal preparations, such as lubricants, recommending that they be mildly acidic (pH 4.5), with an upper limit of osmolality not exceeding 1200 mOsm/kg, to minimise any risk of mucosal/epithelial damage [20,75]. In the context of tonicity, while hypotonic vaginal products enhance muco-penetration, hyperosmolar vaginal products raise safety concerns with respect to vaginal tissue health and sperm viability and mobility [72]. This was corroborated in a phase 1 clinical trial for a vaginal microbicide developed against HIV, which was discontinued, with the sponsor citing unacceptable side effects resulting from the high osmolality of the gel, reinforcing the need to factor in osmolality when developing vaginal semi-solid formulations [168]. In addition, the size of the carriers/particles infused in semi-solid formulations has a direct impact on cervicovaginal mucosal and epithelium penetration, with a 200–500 nm particle size range recommended for VDD mucus [20]. Surprisingly, lowering the particle size range to 100–150 nm results in them becoming trapped and immobilised in the numerous tiny pores/pockets of the cervicovaginal mucosa, rendering them appropriate for localised, deep mucosal drug delivery. In contrast, larger-sized carriers >1000 nm are unable to diffuse into such pores/pockets and so remain on the outer mucosal surface, where they are susceptible to more rapid clearance by ciliated mucosa [20,169].

Considering the biological and physicochemical challenges, nanotechnology-based delivery systems have proven a promising means of improving drug distribution, retention, and therapeutic efficacy in VDD [170]. Nanoparticulate systems can enhance the solubility, bioavailability, and targeting of drugs, while increasing the rates of dissolution and surface area that can be reached. This can be achieved through the design of particulates including, but not limited to, micelles, carbon nanotubes, polymeric lipid nanoparticles, nanocapsules, nanogels, nanofibers, dendrimers, quantum dots, and nanocomposites, which are extensively reviewed elsewhere [157,169,171]. The literature suggests that these nanocarriers have demonstrable drug solubility enhancement properties, while also protecting against rapid drug degradation and enhancing drug concentrations in target tissues, further masking them from the harsh conditions of the FRT and addressing the many shortfalls of conventional VDDS [62,150,151]. For instance, the aqueous solubility, stability, and mucosal permeability of antifungal drugs have been addressed using inclusion complexes and gel flakes [172,173]. Antifungal drugs prepared using hydroxypropyl β-cyclodextrin (HPβ-CD) have been readily incorporated into sol–gel formulations, and exhibited sustained drug release without any detrimental effect on the vaginal tissue [172]. Similarly, enhanced drug permeation/bioavailability was achieved through the vaginal epithelium, alongside improved epithelial drug viability, when in situ nanoparticles of acyclovir, a highly water-insoluble drug, were formed, in comparison to the pure drug [174]. Likewise, atorvastatin, a BCS class II drug, when formulated into nanoparticles exhibited significant improvements in solubility and efficacy, compared to the native powdered atorvastatin [175]. Hence, the use of vaginal sol–gel formulations as vehicles for such tailored and innovative micro- and nano-encapsulated drug forms is a highly promising proposition to address the shortfalls of conventional dosage forms for a range of conditions, as elaborated in Table 4 [1,9,176,177].

The range of gynaecological conditions receiving significant attention through innovative formulation development include vaginal infection and atrophy, neoplasia, labour induction, prophylaxis of HIV, and contraception [72,78,84,106]. Vaginal infections in particular are of growing concern, and, in this context, several studies have focused on the development of sol–gels to treat a range of STIs of bacterial and fungal origin [3,177]. A few studies on vaginal sol–gel systems are represented in Table 4. These studies suggest that the sol–gel formulations, when used in vaginal infections, not only have better efficacy compared to their conventional formulations but also are associated with reduced toxicity towards the underlying tissues of the FRT. Hence, they present strong potential to solve the problems of high reinfection rate and incomplete treatment with the current treatment regimen of vaginal infections. For instance, in a pilot, randomised, controlled trial with confirmed bacterial vaginosis, which has a very high rate of reoccurrence, the initial treatment rate for an in situ gel and gel was 85% and 71.24%, respectively. However, the difference was more prominent with treatment for 4 weeks, with values of 80% with the in situ gel and 47.4% with gel application, indicating the higher long-term efficacy of in situ gels, which can be ascribed to the increased mucoadhesiveness, increased vaginal residence time, and sustained release nature exhibited by the use of poloxamers in in situ gel formulation [177].

Current strategies for HIV prophylaxis utilise vaginal microbicides that act specifically on the critical steps of HIV transmission, and several such formulations are in different stages of development [4,178]. In this regard, vaginal sol–gels provide an opportunity for early intervention to the sexual transmission of HIV in females, with pH-induced gelation triggered by exposure to semen, effectively shielding underlying epithelial cells, and restricting the entry of virions into the systemic circulation [106]. For instance, a pH-responsive polymeric network comprising phenylboronic acid, salicylhydroxamic acid, and 2-hydroxypropyl methacrylamide impeded the migration of HIV at pH ≥ 4.8 [108]. This mechanism gives rise to the concept of “molecular condoms”, where the temperature and pH responsiveness of formulations can be applied to the vaginal mucosa setting, with the gel form effectively covering the mucosal tissue and releasing microbicides [179]. Moreover, this concept of shielding the mucosal tissue helps drug concentration and retention at the vaginal mucosa surface to facilitate mucopenetration and enhance pharmacokinetics at the target tissue [14]. In cases where the microbiome and/or vaginal mucosal tissue integrity is compromised, susceptibility to HIV infection is elevated. Therefore, co-administration of anti-HIV drugs alongside the localised delivery of mucosal barrier formulations is a combination approach to the prophylaxis of HIV infection that warrants widespread use [14]. For instance, thermosensitive nanoparticles of the combination of hydrophilic drug Raltegravir, a HIV integrase inhibitor, and lipophilic drug efaviren (non-nucleoside reverse transcriptase inhibitor), prepared using a poloxamer (Table 5), resulted in thermogelation at 32.5 °C and exhibited anti-HIV activity at a concentration lower than that exhibited by the solution of the combination of these drugs. Moreover, nanoparticles were taken up rapidly by HeLa cells (within 30 min) and exhibited sustained drug release without exhibiting cytotoxicity for a period of 14 days, which indicates that the formulation is a suitable candidate for the prevention of prolonged vaginal pre-exposure of HIV. Furthermore, the incorporation of (RAL + EFV) nanoparticles did not result in the aggregation of nanoparticles, suggesting that the thermosensitive gel is an effective drug delivery vehicle for these anti-HIV drug-loaded nanoparticles [13].

Similarly, intravaginal dendrimer-based sol–gels have also earned considerable attention in recent years for the treatment of the highly challenging HPV infection, particularly in pregnant women, where systemic drug exposure is not desirable for either the mother or the growing foetus [62]. An in situ hydrogel infused with amoxicillin using a generation 4 poly(amidoamine) dendrimer with polyethylene glycol provided in vitro drug release for 240 h and a sustained antibiotic effect through a dual mechanism, i.e., the antibiotic effect of the dendrimer itself and the sustained release of the drug. Moreover, the dendrimer complex targeted the inflammatory cells and reduced cytotoxicity and hence no change in vaginal pH or tissue necrosis was observed while the formulation was retained in the vaginal mucosa (72 h), after which the hydrogel started to become degraded [180].

With these established benefits of VDDS, and the many limitations/unwanted effects associated with current modes of hormonal contraceptive administration, safer and more patient-friendly intravaginal hormonal contraceptives—specifically, stimuli-responsive in situ hydrogels—are gaining attention and interest [78,181]. It has been demonstrated that formulations containing multiple drugs are more efficient contraceptives compared to single-drug formulations [122,181]. In this context, an in situ pH-responsive hydrogel containing indomethacin, gestodene, and ethinyl estradiol prevented pregnancy completely compared to a control group, which presented 100% pregnancy. However, surprisingly, another group receiving hydrogels without any drugs had a 60% pregnancy rate, indicating that the hydrogel components also play a role in contraception [181]. Likewise, in situ hydrogels of non-hormonal agents have also been explored for vaginal contraceptives [10,84]. For instance, a chitosan-based in situ hydrogel of iron (II) gluconate dihydrate, used prior to sexual intercourse, releases iron rapidly in the vagina and exhibits spermicidal properties [10]. In another study, poloxamer-based temperature-responsive in situ hydrogels of nonoxynol-9 resulted in up to 10 h of vaginal residence time [182]. With the rising interest in using multi-drug treatment regimens for more comprehensive therapeutic coverage, drugs with complementary modes of action have been proposed (e.g., anti-HIV + anti- HPV + spermicide), and so the development of sol–gels in this context would be an important milestone in advancing VDD and women’s health and well-being more broadly [14,183]. For instance, vaginal administration of a nanoparticle formulation containing an antimicrobial and spermicidal agent, curcumin, and the anti-HIV agent efavirenz exhibited better encapsulation efficiency compared to single-drug nanoparticles and exhibited better efficacy compared to their solution form, without affecting lactobacilli viability or vaginal tissue, hence indicating the formulation as an efficient example of multiple prevention technology (MPT)-based VDDS [184]. In this context, the development of an efficient delivery vehicle such as sol–gels would enhance the therapeutic benefits [185]. Additionally, the intravaginal administration of hormones infused in sol–gels is also being investigated for hormonal replacement therapy and fertility treatment, while its application in cervical cancer has shown promise (Table 5 and Table 6) [186,187].

**Table 5:** Sol–gel formulations designed for gynaecological indications.

Indication	API	Drug Form	Stimuli-Sensitive and Mucoadhesive Polymers (*w*/*v*)	Gelation Trigger	Gelation Mechanism	Comments	References
Bacterial vaginosis	Metronidazole	Free drug	20% poloxamer 407 and 10% poloxamer 188	Temperature	Swelling due to polymeric crosslinking	Increased prolonged curative rate with sol–gel (80%) compared to conventional gel (47.4%)	[177]
	Clotrimazole	Free drug	15% poloxamer 407, 15% and/or 20% poloxamer 188, and 0.2% *w/v* polycarbophil	Temperature	Micelle formation	Antifungal effect for 10 days; reduced toxicity to epithelium cells of human cervix	[97,158]
	Secnidazole	Aerosol foam	0.45% carbopol 940 with 0.35% HPMC K4 M and 0.35% carbopol 940 with 0.35% HPC	pH	Hydrogen bonding	Less than 50% of drug released by 8 h, indicating controlled drug release	[188]
	Secnidazole	Free drug	20% poloxamer 407, 1% poloxamer 188, and 1 or 2.5% chitosan	Temperature	Micelle formation	Approximately 1–2-fold increase in mucoadhesiveness with chitosan	[11]
	Clindamycin	Free drug	1% gellan gum and 1% HPMC	Ion	Polymeric crosslinking	Good gelling capacity; good mucoadhesion and adequate inhibition of microbial growth	[50,189]
	Voriconazole	Drug- hydroxypropyl β-cyclodextrin inclusion complex	Poloxamer 407, poloxamer 188 HPMC, HEC, polycarbophil, and carrageenan	Temperature	Formation of closely packed micelles in aqueous medium	Increased vaginal tissue uptake by the use of cyclodextrin and sustained drug release for 8 h using in situ gel in female Wistar rats compared to conventional formulation	[176]
	Amphotericin B	Drug- Hydroxypropyl ϒ-cyclodextrin complex	25% poloxamer-based multiblock copolymers	pH and temperature	Hydrogen bonding	Toxicity reduced by complexation; dissolution controlled drug release rate; prolonged drug release observed at pH 7.4 and pH 9.0	[172]
Herpes simplex virus (HSV) infection	Acyclovir	Nanoparticle	18% poloxamer 407	pH and temperature	Polymeric crosslinking	Drug’s therapeutic level achieved with 10 times smaller amount of drug; relative bioavailability increased twice compared to suspension dosage form of pure drug	[174,190]
Infertility	Fetilty-Promoting intrauterine infusion liquid (FPL)	Icariin extracted from Epimedium, safflower, and motherwort	19% poloxamer 407, 2.5% poloxamer 188, and 0.3% HPMC	Temperature	Hydrogen bonding	Uterus and ovarian indices significantly increased in the rats receiving the sol–gel formulation compared to control group; oestradiol levels increased after day 7 to day 22	[191]
	Sildenafil citrate	Free drug	15% poloxamer 407 and 1% HEC	Temperature	Entanglement and condensed micelle packing at increased polymer concentration	Sol–gel transition temperature reduced by addition of HEC; increased endometrial thickness as well as uterine flow with reduced dosing length compared to vaginal suppositories	[192]
Pre- exposure prophylaxis of HIV	Raltegravir + efaviren (RAL + EFV)	Nanoparticles	20% poloxamer 407 and 1% poloxamer 188	Temperature	Hydrogen bonding	Inhibitory concentration of RAL + EFV–NPs less than the solution form; sol–gel proved an efficient delivery vehicle of NPs	[13,193]
	Tenofovir	Microsphere	α,β-glycerophosphate (GP), chitosan, sodium alginate	Temperature	Electrostatic interaction between polymers	Viscosity of chitosan–GP complex strengthened by sodium alginate; initial burst release (30%) in the first 30 min followed by cumulative release (87.82%) after 24 hrs	[194]
Contraceptive	Nonoxynol-9	Free drug	18% poloxamer 407 and 1% or 6% poloxamer	Temperature	Micelle formation	Increased vaginal residence time compared to solution form; rapid hydrogel erosion and drug release	[11,182]
Intrauterine device insertion for contraception	Lidocaine	Free drug	18% poloxamer 407, 5% poloxamer 188, and 0.3% gellan gum	Temperature and ionic strength	Hydrogen bonding between the polymers	Better acceptance and pain management by sol–gel formulation compared to conventional gel	[193]
Hormone replacement therapy, preterm birth	Progesterone	Free drug	5% glycol chitin	Temperature	Hydrophobic interaction	No significant effect on gel property by viscosity reduction after dilution by vaginal fluid but not recommended in presence of semen; prolonged vaginal residence time and controlled drug release	[187,195]
Cervical cancer	Doxorubicin	Free drug	7% glycol chitin	Temperature	Hydrophobic interaction	Initial 20% burst release followed by sustained release for 13 days	[186,195]

HPMC—hydroxypropyl methyl cellulose, HPC—hydroxypropyl cellulose, HEC—hydroxyethyl cellulose.

**Table 6:** Types of polymers used in VDD.

Source	Polymers	Role/Feature	References
Plant	Cellulose derivatives e.g., HPMC, HPC, HEC, MC, EC	Thermo responsive gelation; Mucoadhesive; non-biodegradable	
	Pectin	Mucoadhesive	
	Alginate	Biocompatible; biodegradable; anionic; ion-responsive gelation	
	Carrageenan	Mucoadhesive; antimicrobial and antiviral activity	[50,196]
Animal	Chitosan	Polycationic copolymer; Mucoadhesive; biocompatible; biodegradable; antibacterial activity	[37,50]
	Gelatin	Biocompatible; biodegradable;	[50]
	Hyaluronic acid	Negatively charged	[37]
Microbial	Gellan gum	Ion-responsive gelation	[50]
	Xanthan gum	Form physical gel	[50]
Synthetic	Poloxamers	Non-ionic triblock copolymer; amphiphilic; multi-stimuli responsive gelation	[37,50,197]
	Polyacrylates	Viscosity affected by formulation pH	[37]
	Polyethylene glycol	Water soluble	[50]
	Polyvinylpyrrolidone	Linear; water soluble	[50]

HPMC—hydroxypropyl methylcellulose, HPC—hydroxypropyl cellulose, HEC—hydroxyethyl cellulose, MC—methyl cellulose, EC—ethyl cellulose.

### 4.2. In Situ Sol-to-Gel Phase Transition Stimuli

#### 4.2.1. Thermoresponsive Gelation

**Figure 4:**
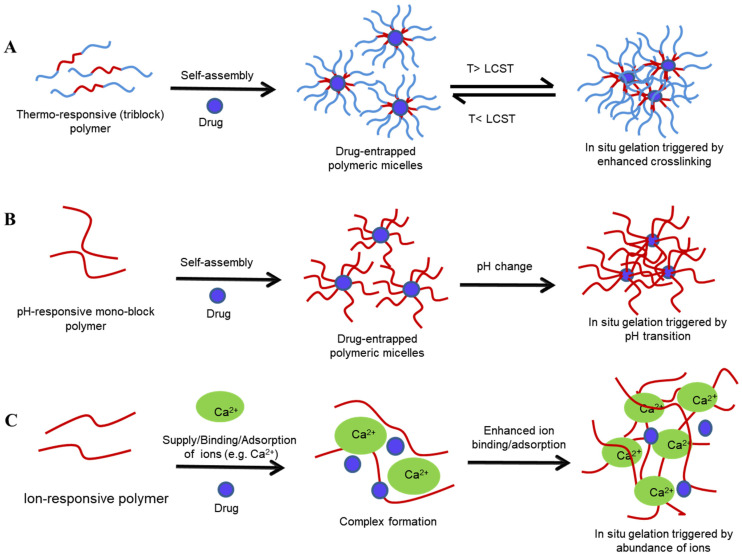
Sol–gel transition of various stimuli-sensitive polymeric systems: temperature-sensitive (**A**), pH-sensitive (**B**), and ion-sensitive (**C**) systems. T—transition temperature, LCST—lower critical solution temperature.

Thermosensitive sol–gel systems comprise polymers that undergo gelation at/approaching body temperature. Gelation occurs physically by the entanglement of polymer chains, micelle packing due to self-assembly of the polymeric micelles at elevated temperatures, physical crosslinking due to the dehydration of the polymeric block above a lower critical solution temperature (LCST), hydrophobic interaction, and the transition of a coil into helix form [6,8]. The aqueous solutions of thermogelling polymers have a perfect balance of hydrophilic and hydrophobic groups, which is disturbed with the slightest change in temperature, and they undergo phase separation at the critical solution temperature (CST) [8,158]. For the polymers exhibiting LCST, phase separation occurs above CST, whie the opposite is true for polymers exhibiting an upper critical solution temperature (UCST) [198]. The hydrophilic polymers become hydrophobic and insoluble above their LCST, resulting in gel formation (Figure 4A). LCST determines the thermo-reversibility of thermoresponsive systems and depends upon the polymer concentration [8]. There is an inverse relationship between polymer concentration and gelation temperature, driven by the hydrophobic force [8,199]. At a higher polymer concentration, hydrophobic interaction increases due to molecular crowding, resulting in gelation at a lower temperature [190]. Polymers are typically used in concentrations that trigger gelation in the 25–37 °C range, in the context of VDD. Using an appropriate applicator (discussed later in Section 5), an appropriately engineered sol–gel can provide ease of application, while its rapid transformation to a viscous gel can reduce leakage, enhancing retention on the vaginal mucosa [6]. Here, a gelling temperature close to the physiological temperature is ideal for the stabilisation, solubilisation, and controlled release of hydrophobic drugs, as the polymeric monomers aggregate to form micelles within their hydrophobic core, wherein the solubilised hydrophobic drug resides [158,162]. The concentration of thermogelling polymers, co-solutes, and dilution by fluid in the vagina affect the gelation temperature and the viscosity of the gel formed. Hence, it is essential to characterise thermosensitive systems in simulated conditions to help predict their in vivo performance [8,97]. Temperature-stimulated sol–gel transition is a commonly employed phenomenon in several studies of VDDS, even though dual stimuli are also employed for sol–gel transition (see examples in Table 5).

- Poloxamers

Poloxamers (Pluronic^®^) are triblock copolymers of poly(ethylene oxide)-poly(propylene oxide)-poly(ethyleneoxide) (PEO-PPO-PEO) units. They are amphiphilic in nature, with two outer hydrophilic PEO segments and an inner hydrophobic PPO segment that can partly solubilise hydrophobic drugs [190]. However, such characteristics result in inconsistent drug release profiles since the drug loaded in the PEO portion is released prior to gel dissolution, in contrast to the drug loaded in the PPO portion, which is released after gel dissolution; hence, modification of the formulation is required for better drug release characteristics [197]. An increase in temperature causes a change in the orientation of the methyl group of the side chain and dehydration of the PPO segment, as well as water extrusion from the micellar core of poloxamers, resulting in gelation [158]. Above the critical micellar concentration of polymers (CMC), the hydrophobic cores of the micelles absorb water and can also accommodate and solubilise hydrophobic drugs [158]. This encapsulation process can also protect drugs from cellular interactions and degradation [200]. Although poloxamers are water-soluble at room temperature and have excellent gelling properties at body temperature, their lack of inherent mucoadhesiveness warrants the use of mucoadhesive polymers (e.g., chitosan, carbopol, HPMC, which are discussed below), although their addition can disrupt the gels’ rheomechanical properties; thus, further fine-tuning of the poloxamer composition is usually warranted [11,162,201]. For instance, supplementation of poloxamer 407 with poloxamer 188 increases the mechanical strength of the gels and hence slows polymer erosion and modulates drug release. On the other hand, the higher hydrophilicity of poloxamer 188 can result in increased polymer erosion and rapid drug release, thus balancing the concentrations, and tailoring them to the infused drug is needed to ensure optimal mucoadhesion, polymer erosion, and drug release [182].

- Cellulose derivatives

MC and HPMC exhibit thermoresponsive behaviour at 40–50 °C and 75–90 °C, respectively [202]. Gelation occurs by polymer–polymer hydrophobic interactions at higher temperatures due to the loss of incomplete but sufficient water for the hydration of the polymers, leading to the association of polymer units and gel formation. When the temperature of these polymers is increased, the viscosity of the polymers is reduced, which, on further heating, increases again, driving gel formation [31,203]. However, the gelation temperature can be reduced by the use of physical and chemical methods—for example, the addition of NaCl to MC solution reduces its transition temperature to 32–34 °C [161]. In the context of VDD, ethyl(hydroxyethyl) cellulose, whose viscosity is reduced on increasing temperature, has a reverse character after incorporating an ionic surfactant such as sodium dodecyl sulfate, cetyl triammonium bromide, etc., and undergoes gelation at a temperature of 30–40 °C, making it a suitable polymer for VDDS [158,161].

- Gelatin

Gelatin forms a gel when the temperature is lowered, due to the conversion of coils into helices through hydrogen bonding as well as van der Waals forces, and hence is grafted with other polymers to ensure the desired sol–gel transition in the human body [204]. For instance, gelatin combined with poly-*N*-isopropylacrylamide produces a thermoresponsive matrix, which undergoes rapid gelation at 37 °C [8].

#### 4.2.2. pH Sensitive Sol–Gel Systems

Here, polymers contain weakly acidic or basic groups capable of donating or accepting H^+^ ions depending upon the environmental pH, leading to the ionisation, association, and binding of ions to the polymer chains, resulting in changes in polymer conformation and solubility, both of which are drivers of gelation (Figure 4B) [159,205]. Such changes occur at a specific pH known as the transition/critical pH and it depends upon the pKa of the polymer [1,159]. pH-responsive delivery is a promising approach for the delivery of poorly water-soluble drugs such as paclitaxel, for the treatment of ovarian and cervical cancer. Here, the elevated pH of tumour cells triggers the release of chemotherapeutic agents from the drug formulation containing the pH-responsive polymer mPEG2000-Isopropylideneglycerol [206]. pH-responsive gelation has also been employed in the prophylaxis of STIs and HIV, wherein drug activity is delayed by the vaginal pH and only triggered in the presence of a higher pH once semen is detected [159]. Human semen, with a pH of 6.5 to 7.0, has a high buffering capacity and hence acts a trigger for gelation and drug release, resulting in the inactivation of HIV or other pathogens. The resulting gel acts as a protective microbicide, coating virus particles at the vaginal epithelium, although a short mucosal residence time usually warrants co-formulation of the gel with mucoadhesive polymers [14,159].

- Chitosan

Chitosan, a naturally derived glucosamine and *N*-acetylglucosamine polymer, is widely used in the pharmaceutical sector owing to its cationic-based mucoadhesiveness and antimicrobial activity [9,166]. The positively charged groups in chitosan interact with the negatively charged mucin layer, developing a strong attractive force resulting from the hydrogen bonding, coulombic force, and hydrophobic interactions between chitosan and mucin. Prolonged adhesion of chitosan gels in the vaginal mucosa results in sustained and comprehensive drug release, wherein it disrupts intracellular junctions on the vaginal mucosa, providing mucopeneterating characteristics [166]. It promotes gelation in the pH 6–7 range due to deprotonation of the amine groups, which is an advantage for VDD. However, chitosan also becomes insoluble in the basic pH range, which presents practical challenges to its use in sol–gel systems [207]. Interestingly, the pH sensitivity of chitosan systems can be transformed into a thermosensitive nature by supplementation with polyol salts [161,166]. For instance, a combination of chitosan and alginate at the ratio of 1:2 *w*/*w* provides an improved antibiotic effect and better control of drug release compared to the use of chitosan alone [208]. Due to such features, chitosan has been widely employed in formulations for treating vaginal infections. Furthermore, it has been found that microparticles prepared using chitosan effectively encapsulate both hydrophilic and hydrophobic drugs for VDD, paving the way for multi-drug delivery [148,166].

- Polyacrylates (PA)

PAs are esters of acrylic and methacrylic acids and are commercially available as Eudragit^®^, Kollicoat^®^, and Eudispert^®^ [50]. Carbopol and polycarbophil are the most commonly used PAs for VDDS and are found to be effective for both local and systemic effects [37]. A drawback, however, includes the limited drug loading capacity for poorly aqueous soluble drugs [209]. Carbopol is highly versatile, serving as a mucoadhesive agent, viscosity modifier, and hydrophilising agent in various liquid and semi-solid formulations for VDDS [50]. Phase transition of carbopol occurs when the pH increases beyond its pKa value of 5.5. In the acidic environment of the vagina, the carboxylic group of carbopol dissociates, resulting in increased intra-polymeric ionic repulsion, which causes swelling of the uncoiled polymeric chain, eventually forming a completely packed gel structure [158,210]. The mucoadhesive nature of carbopol is ascribable to its ability of forming hydrogen bonds with mucin of the vaginal mucosa [211]. Polycarbophil is found to possess a normalising effect on the vaginal pH during menopause and vaginitis, and is often employed as the mucoadhesive polymer of choice [7].

#### 4.2.3. Ion-Sensitive Sol–Gel Systems

Anionic polysaccharides, which undergo gelation by crosslinking in the presence of ions, are employed to create ion-sensitive systems [1]. Here, the solution forms of drug–polysaccharide complexes undergo gelation in the presence of ions existing in vaginal fluid, most typically sodium (Na^+^), calcium (Ca^2+^), potassium (K^+^), and chloride (Cl¯) [2]. Although limited studies have been published that use ion-responsive systems, they provide another avenue for investigation to circumvent the shortfalls related to conventional formulations for VDD.

- Gellan gum

Being an anionic polymer, gellan gum undergoes gelation via hydrogen bonding between the ions and water through the formation and subsequent aggregation of double helical structures in the presence of monovalent, divalent, and trivalent ions [2,53]. The role of cations is crucial during this process and divalent cations are found to have greater gelling capacity than monovalent cations [2]. Vaginal sol–gel formulations of clindamycin have been prepared using gellan gum and supplemented with HPMC, the latter of which aligned the gelation temperature of gellan gum close to body temperature, providing a well-tolerated formulation and a viable alternative to conventional VDDSs [189].

- Alginate

Alginate is an acidic polysaccharide that contains residues of (1,4)-linked *β*-D-mannuronate (*M*) and *α*-L-guluronate (*G*), undergoing gelation on binding with divalent (e.g., Ca^2+^) and trivalent (e.g., Al^3+^ and Fe^3+^) ions [207]. Ions drive the dimerisation of two *G* chains oriented in opposite directions, forming a hydrophilic cavity, serving as the binding site for ions, while each ion is capable of binding four *G* chains. The resulting orientation resembles an “egg-box”, and an interconnecting gel network forms, resembling a “zip” (Figure 4C) [212]. Sodium alginate, when used in a thermosensitive polymeric microsphere of tenofovir, did not impact gelation time; however, it strengthened the gel, supporting adherence to the vaginal mucosa and resulting in extended drug absorption kinetics [194].

- Pectin

Pectin is another polysaccharide, consisting of methoxylated galacturonic acid units, with gelation related to the degree of methoxylation; low methoxylation content is desirable for appropriate responses to ionic changes [158]. A pseudo-“egg-box” model has been proposed as a gelation mechanism of pectin wherein Ca^2+^ ions bind to the antiparallel pectin chains, forming egg-box dimers [213]. Studies using pectin for localised VDD of fungistatic/fungicidal agents have shown promise and warrant further clinical investigation [37,214].

## 5. Applicators for Intravaginal Administration of Dosage Forms

The effectiveness of VDDSs is largely influenced by the patients’ acceptance and adherence to treatment regimens, which is ultimately determined by the overall user experience. Acceptable user experience can be achieved by ensuring ease of use and patient comfort when administering any vaginal product [215]. Applicators make the vaginal drug administration convenient and drug delivery more reliable. They are classified as class I medical devices and hence possess low risk to the user and are subjected to minimal regulatory control [216]. Although vaginal products can be administered without an applicator, studies suggest the preference of an applicator, despite the associated elevated costs to patients/consumers. Moreover, the physical attributes of the applicator, including the length, width, colour, comfort, ease of grip and use, overall appearance, and environmental friendliness, have been found to influence the choice of applicator [215,216].

Generally, applicators are an optional tool for administering solid dosage forms such as tablets and capsules. However, their use becomes critical when administering liquids, semi-solids, and foams, which typically require deep insertion of the formulation, and applicators offer the advantage of more uniform drug distribution and localised targeted delivery while mitigating leakage and systemic effect [3,19]. Semi-solid formulations, such as creams and sol–gels, need to be sufficiently free-flowing to be used in syringe applicator-based devices, so that the formulation can be ejected via a plunger with ease [49]. Historically, vaginal applicators were developed to deliver contraceptives to the cervix and hence drug exposure to the entire vaginal mucosal tissue was not considered critical [217]. However, increasingly so, the focus has shifted more towards the development of vaginal microbicides, wherein the applicator’s role has become more critical in ensuring delivery to a larger proportion of the lower FRT [217]. As a result, device manufacturers have designed applicators with pores along their length, which ensures that the formulation spreads across a larger surface area of the vaginal mucosa when actuated; this is in contrast to the delivery profile of conventional applicators that aim to deliver the drug into the cervix and upper FRT [6,217]. Recently, a non-hormonal contraceptive with a pre-filled applicator and multiple-pore design was approved by the U.S. FDA, providing on-demand contraception when used 1 h before or immediately after sexual intercourse [123]. Similarly, dinoprostone is used to induce labour and is administered deep in the endocervical canal using an applicator inserted intravaginally by trained personnel [19].

The lack of a suitably designed applicator can seriously hamper the effectiveness of even the best therapeutics, and so patient experience/acceptability must go hand in hand with dosage form and applicator design if expected clinical outcomes are to be met [210]. Selection of a suitable applicator design for VDD has historically been somewhat of an afterthought, although the tide is turning with new vaginal applicators on the horizon, some of which are highlighted in Table 7.

## 6. Conclusions and Future Perspectives

Several studies demonstrate the wealth of opportunities offered by the vaginal route in the treatment of multiple local and systemic gynaecological conditions, including STDs/STIs, contraception, HRT, infertility, and cancer. However, the highly variable anatomical and physiological features of the FRT make the therapeutic outcome highly challenging using currently available conventional methods of VDDSs. This has led to an increased demand for novel drug delivery techniques that are capable of addressing the limitations of conventional VDDSs, including vaginal leakage, lower drug residence time, and lower patient compliance and adherence to the treatment regimen. In this context, sol–gel technology, through the utilisation of “smart” polymers possessing stimuli-responsive and mucoadhesive characteristics, plays a promising role in the safe and efficient delivery of diverse drug molecules in a controlled and sustained manner. In addition, this formulation technology may be a carrier for other novel drug delivery platforms including microencapsulation, nanotechnology, liposomes, micro-emulsions, inclusion complexations, and drug-eluting fibres to overcome their limitations raised in the absence of suitable drug delivery vehicles, resulting in the successful delivery of drug molecules of diverse nature, which would otherwise have not been delivered effectively using conventional formulation approaches. Hence, the sol–gel formulation approach addresses the limitations of conventional formulations by taking advantage of recent advances in drug delivery technology. Although the sol–gel formulation approach is in the early stage of development, the studies performed in the past decade strongly demonstrate its advantages in mitigating a range of gynaecological conditions that are affecting the health and overall quality of life of more than half of the world’s population. Recent studies on sol–gel formulations are focused on the use of microbicides and contraceptives. Unfortunately, none of these formulations are in clinical study or use yet, suggesting the need for more studies on this aspect. However, the brighter side is that such formulations could be expected in the near future considering the number of studies conducted and the benefits offered by these formulations. Moreover, future studies on drug delivery to the entire FRT to treat infertility and neoplasia, as well as in conditions requiring hormone delivery, using sol–gel formulation strategies can also be expected.

## Figures and Tables

**Table 7:** Summary of vaginal applicators used in clinical practice.

Applicator Type	Dimensions (mm)	Features	Advantages	Disadvantages	Product Examples	Reference
Single use	114 × 12.7 with a tapered, rounded tip	Comprises plunger, barrel, and cap fabricated from PP and a piston inside the barrel made of non-latex rubber; pre-filled or manual filling	Reduced cost due to bulk production	Higher plastic waste	KY-gel; Canesten^®^ cream	[217,218,219]
Multiple use	114.5 × 11.3	Comprises barrel and plunger fabricated from PE	Can be refilled and reusable, reducing packaging, storage, and transportation costs	Sanitary concerns	Ovestin^®^ intravaginal cream	[215,218,219]
Single-use squeeze tube	105 × 29 tube, plus 5-mm-wide applicator tip	Single-piece device fabricated from PE	Pre-filled, cost-effective	Cannot be filled manually	Norden-Pac applicator	[218]
Multiple pores	-	Presence of PE-fabricated membrane around the reservoir, infused with drug product and with perforations	Covers entire vaginal mucosa immediately after application; uniform drug delivery; pre-filled; biodegradable	High manufacturing cost	Universal vaginal applicator	[217]

PP—polypropylene, PE—polyethylene.
